# Evaluation of Black Soldier Fly *Hermetia illucens* as Food for Pink-Spotted Lady Beetle *Coleomegilla maculata*

**DOI:** 10.3390/insects14120902

**Published:** 2023-11-22

**Authors:** Eric W. Riddick, Ryan C. Walker, Maria Guadalupe Rojas, Juan A. Morales-Ramos

**Affiliations:** National Biological Control Laboratory, Agricultural Research Service, United States Department of Agriculture, Stoneville, MS 38776, USA; ryan.walker2@usda.gov (R.C.W.); guadalupe.rojas@usda.gov (M.G.R.); juan.moralesramos@usda.gov (J.A.M.-R.)

**Keywords:** augmentative biological control, *Artemia franciscana*, *Chlorella vulgaris*, Coccinellidae, factitious diet, mass rearing, myristic acid, Stratiomyidae

## Abstract

**Simple Summary:**

The discovery of new and improved diets is necessary to mass rear predators of high quality to support the biological control of plant pests on crop plants. This study evaluated the black soldier fly (BSF) as an alternative food source for mass rearing of the pink-spotted lady beetle, which is a predator of aphids. The hypothesis that BSF larval powder supported the growth, development, and reproduction of the predator was tested in the laboratory. When compared to a standard in-house diet containing brine shrimp egg powder plus algae and myristic acid (BSE+CM), the BSF diet reduced immature growth and development. Immatures successfully reared to adults were smaller when reared on BSF or BSF+CM. Combining BSF with an artificial diet (AD) in a 50:50% ratio (i.e., BSF+AD) did not improve predator growth or development. Predator oviposition responses to BSF versus BSE+CM or BSF+AD versus BSE+CM did not differ significantly. In conclusion, BSF has the potential to be food that supports predator oviposition behavior.

**Abstract:**

The discovery of new and improved factitious and artificial diets is necessary for cost-effective rearing of predatory arthropods. This study evaluated *Hermetia illucens* black soldier fly (BSF) as a suitable alternative food source for rearing the predatory coccinellid *Coleomegilla maculata* (*Cmac*). The hypothesis that BSF larval powder was suitable food to support the growth, development, and reproduction of *Cmac* was tested in the laboratory. When compared to a standard in-house diet containing brine shrimp egg powder plus *Chlorella vulgaris* green algae and myristic acid (BSE+CM), the BSF and BSF+CM diets reduced immature growth and development. Immatures successfully reared to teneral adults were smaller when fed BSF or BSF+CM rather than BSE+CM. Combining BSF with a powdered artificial diet (AD), i.e., BSF+AD, did not improve predator growth or development, compared to *Cmac* reared on BSE+CM. *Cmac* oviposition responses, i.e., egg clutch production, to BSF vs. BSE+CM or BSF+AD vs. BSE+CM did not differ significantly. In conclusion, BSF has the potential to be food that supports *Cmac* oviposition behavior. Future research is necessary to discover an ideal mixture of BSF, BSE+CM, or AD that supports *Cmac* growth, development, and reproduction over multiple generations.

## 1. Introduction

To promote the use of biological control as an alternative to pesticides, new and improved technologies are necessary to mass produce natural enemies, including predators and parasitoids at a reasonable cost [1,2]. Research to discover technologies to mass rear predators has been ongoing for decades, with limited success [3]. There remains a desperate need to discover more cost-effective factitious foods and artificial diets to produce the large quantities of predators necessary to support augmentative biological control [4]. Advancements in the production of coleopteran predators have been compiled recently [5], and more recent work has continued with the aim of discovering more effective diets via the utilization of lepidopterans, dipterans, crustaceans, and juvenile hormones [6,7,8,9,10,11].

A recent development in the feed industry has involved the large-scale commercial production of the black soldier fly (BSF) *Hermetia illucens* (L.) (Diptera: Stratiomyidae) [12,13,14]. It has enormous potential to revolutionize agriculture and aquaculture because larvae digest wastes of plant and animal origin, converting them into usable protein and fats (lipids) [15,16,17,18]. Moreover, BSF larvae could provide an alternative source of protein and lipids, e.g., to replace fishmeal, in diets for farm-raised invertebrates, e.g., shrimp, and vertebrates, e.g., fish [19,20,21]. 

Protein and lipids from the BSF could potentially be used in diets to mass-produce invertebrate predators in support of the biological control industry. In one study, supplementing up to 20% of a yeast extract and hen’s egg yolk-based artificial diet with hemolymph from BSF resulted in a shortened development time and enhanced reproductive capacity of the phytoseiid mite *Amblyseius swirskii* (Athias-Henriot) [22]. This study suggests that BSF hemolymph contains nutrients that can improve artificial diets for predatory mites. No other studies have tested the effects of BSF hemolymph or other body components in artificial diets or as a standalone factitious food for predatory mites. No studies have tested the potential of BSF to support the growth, development, or reproduction of predatory insects, such as lady beetles (coccinellids).

The pink-spotted lady beetle *Coleomegilla maculata* DeGeer (*Cmac*) (Coleoptera: Coccinellidae) is distributed in agricultural landscapes in North, Central, and South America [23,24,25]. It is a predator of aphids (Hemiptera: Aphididae) and other soft-bodied insects [26,27,28]. It also has a proclivity for consuming plant pollen [29,30]. *Cmac* has been reared continuously in our laboratory for more than a decade, using a factitious diet based on brine shrimp *Artemia franciscana* Kellogg (Anostraca: Artemiidae) decapsulated egg (i.e., BSE) powder, *Chlorella vulgaris* Beijerinck (Chlorellales: Chlorellaceae) green algal powder, and a fatty acid, e.g., palmitic acid. To expand our knowledge of the factitious food or artificial diet spectrum for rearing *Cmac*, this investigation considered the BSF as an inexpensive, readily available, protein and fat-rich food source for *Cmac*. BSF protein and fat content were 42% and 22%, respectively, when reared on spent barley grains, which were supplemented with Brewer’s yeast [31]. In this study, the hypothesis that the BSF could be used as a food source to replace more expensive factitious foods or function as a supplement in artificial diets was tested.

## 2. Materials and Methods

### 2.1. Insect Colonies

Two separate *Cmac* colonies were reared continuously in separate environmental rooms (24–25 °C, 16 h:8 h L:D, and 45–55% RH) for more than a decade at the National Biological Control Laboratory (NBCL), ARS, USDA, in Stoneville, MS, USA. Both colonies originated from individuals collected by ARS, USDA colleagues near Beltsville, MD, USA. One colony (i.e., BSE-reared colony) was fed an in-house factitious diet containing a 90:5:5% (dry weight) BSE powder, green algae *C. vulgaris*, and a fatty acid, e.g., palmitic acid, respectively [32,33]. Palmitic acid has been identified in tissues of overwintering *Cmac* [34] and tissues and cornicle secretions of aphids [35,36]. The other colony (i.e., AD-reared colony) was fed an in-house artificial diet (AD) based on protein derived from yellow mealworm, *Tenebrio molitor* L. (Coleoptera: Tenebrionidae) pupal powder, and a mixture of other components [37]. Both colonies had not received any wild-type (feral) individuals since their inception.

### 2.2. Experimental Design and Diet Treatments, Experiment 1

Experiment 1 was designed to evaluate the effects of BSF larval powder on the growth, development, and early oviposition responses of *Cmac* from the BSE-reared colony. This experiment consisted of the following diet treatments: brine shrimp egg (BSE), powder plus *C. vulgaris* algae (C), and myristic acid (M) (BSE+CM) in a 90:5:5% dry weight mixture, BSF larval powder alone (BSF), and BSF+CM in a 90:5:5% dry weight mixture. BSE was purchased from Brine Shrimp Direct Inc. (Ogden, UT, USA; www.brineshrimpdirect.com, accessed on 24 October 2023) and stored in a laboratory freezer. The crude protein and fatty acid content in the BSE were 53.6% and 7.3%, respectively, based on the product label. Eggs were milled into a fine powder formulation using a Waring^®^ 1 L blender (A. Daigger & Company Inc., Vernon Hills, IL, USA; www.daigger.com, accessed on 24 October 2023). *Chlorella vulgaris* green algae powder was purchased from ZNatural Foods (West Palm Beach, FL, USA; www.znaturalfoods.com, accessed on 24 October 2023) and stored in a laboratory refrigerator. The crude protein and fat content in *C*. *vulgaris* were 4% and 0%, respectively, based on the product label. Combining *C*. *vulgaris* with a “synthetic pollen” restored *Cmac* fecundity in experimental arenas provisioned with unsuitable prey, the tetranychid *Tetranychus urticae* Koch [38]. Myristic acid powder (product no. 70082, ≥98% purity, GC grade) was purchased from Sigma-Aldrich Corporation (St. Louis, MO, USA; www.sigmaaldrich.com, accessed on 24 October 2023) and then stored in a chemical cabinet at room temperature. When incorporated into a casein or yeast-based artificial diet, myristic acid enhanced the growth, development, and reproduction of the coccinellid *Olla abdominalis* (Say), syn., *v*-*nigrum* (Mulsant) [39,40]. BSF larval meal was purchased from EVO Conversion Systems (College Station, TX, USA; www.evoconsys.com, accessed on 24 October 2023) and then stored in a laboratory freezer (−20 °C). Note that BSF larvae were reared on a mixture of spent grain and bread waste at EVO Conversion Systems. The crude protein and fat content in BSF larvae were not listed on the product label. At NBCL, BSF larval meal was removed from the freezer and milled into a coarse powder using a Waring^®^ blender prior to experimentation. The unused powder was kept in the freezer until time for experimentation.

To evaluate the effects of diet treatments on *Cmac* growth and development, first instar larvae were harvested at random from egg clutches from the same generation and deposited by mated females onto facial tissue paper in oviposition cages [32,33] in the NBCL colony. Medium-sized Petri dish arenas (clear plastic, 159 cm^3^ volume, 9.0 cm wide, and 2.5 cm high) were used to randomly separate 10 first instars into each arena. Ten replicate arenas were used for each treatment. This resulted in a total of 100 first-instar larvae per treatment diet and 300 first instars in the experiment. Each arena was supplied with at least 30–40 mg of treatment diet (which exceeded the quantity of diet that 10 early instars could consume in several days) at the base, and a small glass vial, stoppered with cotton, provided distilled water for developing larvae. The diet quantity was increased to approximately 80 mg per arena for older instars. Larval growth and development were monitored daily. The old diet was replaced with a fresh diet each week. Cast exuvia and waste products were removed from the arenas as needed. The experimental arenas were held in the same location within the environmental room (24–25 °C, 16 h:8 h L:D, and 45–55% RH) that housed the *Cmac* colony reared on the in-house factitious diet, BSE+CM.

The time (in days) to metamorphose into pupae, pupal survival, and adult survival were recorded. Teneral adults were removed within 24 h of emergence from pupal skins and weighed (to the nearest mg) using a Sartorius analytical balance (Model Entris^®^ BCE124-1S; Sartorius Company, Göttingen, Germany, www.sartorius.com, accessed on 24 October 2023). A total of 87, 52, and 61 teneral adults reared from BSE+CM, BSF, and BSF+CM diets, respectively, were weighed in Experiment 1. To prevent bodily harm to the adults, the adult sex ratio was not determined. Adults were placed in clear plastic cages (500 mL, 7 cm tall, 10.5 cm wide, with screened lids) to observe “first” mating (*in-copula*) behaviors amongst males and females fed a fresh diet of the same treatment given to larvae. Therefore, a minimum of three cages, one per diet treatment, was used to observe the first mating. Distilled water in a stoppered glass vial was provided at the base of each cage. The time (in days) from emergence to first mating was recorded. On the same day, mating pairs were placed into separate oviposition cages (clear plastic, 473.2 mL, 9.5 cm tall, 7.0 wide, with screened lids), with one mating pair per cage. A tissue paper substrate was placed in each cage to serve as an oviposition substrate. A small food dish (1.0 cm tall, 3.5 cm wide) containing 50–60 mg of the treatment diet was positioned at the base of each cage. A glass vial with distilled water was placed at the base of each cage. Any remaining diet was replaced with a fresh diet each week; accumulated waste was also removed. Oviposition responses of mated females were determined within a 30-day evaluation period from the date of placement into oviposition cages. Females preferentially oviposited onto the tissue paper, but occasionally also on the wall or underside of the cage lid. Oviposition cages were checked daily for egg clutches. They were promptly removed on the same day, and the date from first mating to the presence of the first egg clutch was recorded. Also, the number of eggs in each clutch was recorded. The egg hatch rate was not determined.

In summary of this section, 10 replicate arenas, containing 10 first instars, were established on the same day for each treatment, BSE+CM, BSF, and BSF+CM. Therefore, 300 *Cmac* first instars were involved in evaluating diet effects on growth and development. The Petri dish arena served as the sampling unit for statistical analyses. For diet effects on *Cmac* oviposition responses, 13, 7, and 8 females (with mates) were placed in replicate cages provisioned with BSE+CM, BSF, or BSF+CM diets, respectively. Thus, 28 mated females were tested in this experiment. The oviposition cage was the sampling unit for statistical analyses. 

### 2.3. Experimental Design and Diet Treatments, Experiment 2

Experiment 2 was also designed to evaluate BSF larval powder on the growth, development, and early oviposition responses of *Cmac* from the BSE-reared colony. Treatment diets consisted of BSE+CM and BSF+AD (black soldier fly larval powder plus an artificial diet, in a 50:50% *w*/*w* ratio). The artificial diet (AD) was a modification of one mentioned previously [37]. It was devoid of any insect (mealworm) protein or fat components. The BSF was intended to replace the mealworm components. In this AD, hen’s egg yolk and soy lecithin accounted for approximately 20% of the protein and 8% of the fat, respectively [37]. The other aspects of the experimental design and protocols in Experiment 2 were identical to Experiment 1. Note that 87 and 55 teneral adults reared from BSE+CM and BSF+AD diets, respectively, were weighed in Experiment 2. Also, the number of mated pairs involved in the section on diet effects on oviposition responses was different in Experiment 2. In this experiment, 12 and 9 females (with mates) were placed in replicate cages provisioned with BSE+CM or BSF+AD diets, respectively. Thus, 21 mated females were tested in this experiment. The Petri dish arena represented the sampling unit in the test of diet effects on *Cmac* growth and development. The oviposition cage was the sampling unit in the test of diets on *Cmac* oviposition responses. 

### 2.4. Statistical Analysis

The one-way analysis of variance (ANOVA) and Student’s *t*-test were used to test the significance of diet treatments on the immature growth, development, and oviposition responses of *Cmac* in Experiment 1 and Experiment 2, respectively. Mean values were considered significantly different when *p* < 0.05. Prior to subjecting data to ANOVA or Student’s *t*-test, a normality test (Shapiro–Wilk) and an equal variance test (Brown–Forsythe) were conducted. Following the ANOVA, the Holm–Sidak multiple comparison procedure was used to separate mean values if significant differences were detected. The Pearson Product-Moment Correlation, with statistic *r*, was used to test for significant correlations between days to pupal stage versus adult body mass, emergence to mating (days) versus egg clutch production, and onset of mating to first clutch (days) versus egg clutch production. Correlations were considered significant when *p* < 0.05. SigmaStat^®^ interfaced through SigmaPlot^®^ for Windows V.15 (©2023, Systat Software Inc., San Jose, CA, USA) and JMP^®^ 17.0.0 (©2022, JMP Statistical Discovery, Cary, NC, USA) software were used for data analysis.

## 3. Results

### 3.1. Experiment 1

**Figure 1:**
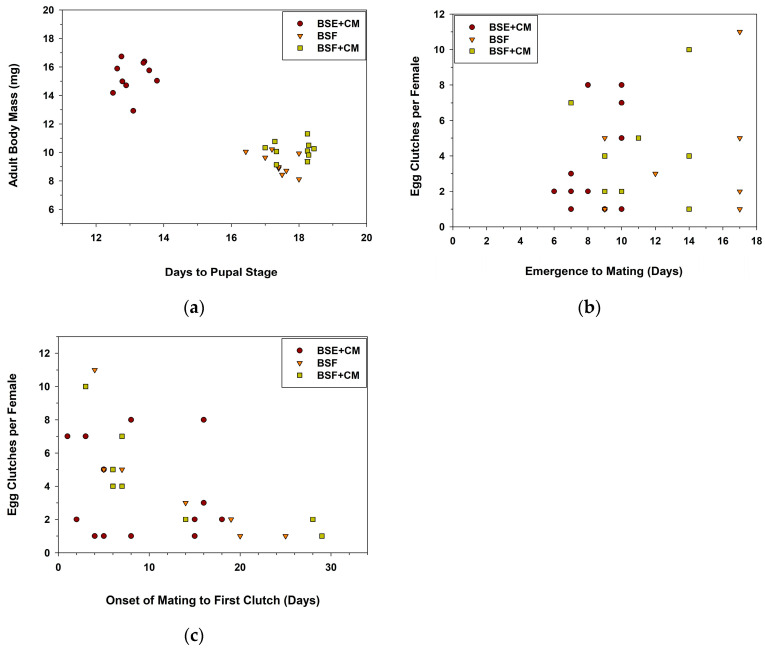
(**a**–**c**) Scatterplots of days for *Cmac* to metamorphose into pupae versus adult body mass (**a**), days from adult emergence to mating versus the number of egg clutches per female (**b**), and the onset of mating to the first egg clutch oviposited over a 30-day time frame (**c**). Diet treatments included brine shrimp egg powder plus *Chlorella vulgaris* algae and myristic acid (BSE+CM), black soldier fly larval powder (BSF), or a novel mixture (BSF+CM). See Table 2 for complementary data.

The BSF and BSF+CM diets were less effective than the BSE+CM diet in supporting *Cmac* immature growth and development (Table 1). *Cmac* larvae took significantly longer to metamorphose into pupae; fewer larvae survived to the pupal stage. Similarly, fewer pupae metamorphosed into adults, and teneral adults weighed less when reared on the BSF diets (Table 1). Irrespective of diet, the days required for *Cmac* larvae to metamorphose into pupae was negatively correlated with the live body mass of emerged adults (Figure 1a; *r* = −0.912, *p* < 0.0001, *N* = 30); longer development time correlated with the emergence of smaller-sized adults.

The time between adult emergence (in days) until *Cmac* adults began mating was affected by diet (Table 2). Adults fed BSF took longer to mate (i.e., *in-copula* pairing of males and females) than adults fed BSE+CM; no differences were detected between those fed BSF and BSF+CM. However, the time between emergence and mating was not correlated with oviposition responses of *Cmac* females, i.e., the production of egg clutches (Figure 1b; *r* = 0.22, *p* = 0.249, *N* = 28). The time from the onset of mating to the production of the first egg clutch did not differ significantly amongst diet treatments (Table 2). Irrespective of diet, the onset of mating to the production of the first egg clutch was negatively correlated with total clutch production per female within the 30-day evaluation period (Figure 1c; *r* = −0.516, *p* = 0.005, *N* = 28). Diet had no effect on the number of clutches produced by *Cmac* females (Table 2). However, diet affected the number of eggs within a clutch; females fed BSF produced fewer eggs per clutch than those fed BSE+CM. No differences were detected between females fed BSF and BSF+CM. 

### 3.2. Experiment 2

**Figure 2:**
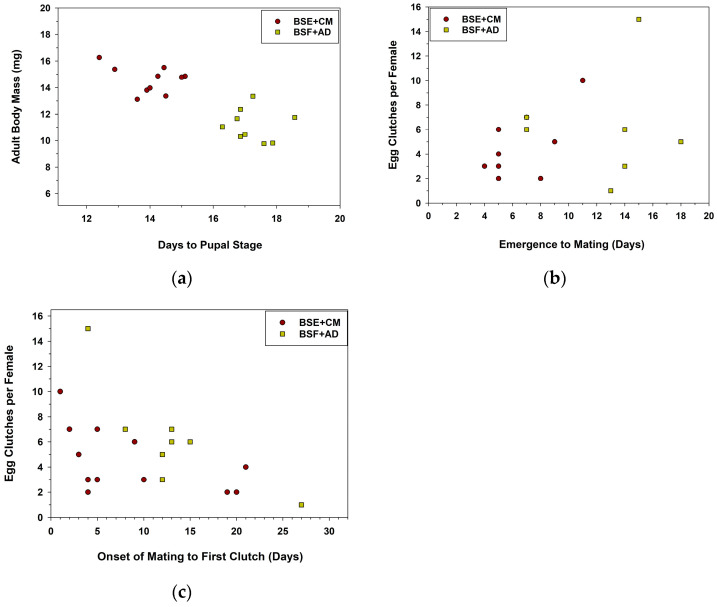
(**a**–**c**) Scatterplots of days for *Cmac* to metamorphose into pupae versus adult body mass (**a**), days from adult emergence to mating versus the number of egg clutches per female (**b**), and the onset of mating to the first egg clutch oviposited over a 30-day time frame (**c**). Diet treatments included BSE+CM, and a novel mixture of black soldier fly plus an artificial diet (BSF+AD); the sample size was 12 and 9 females (paired with one male) in respective treatments in oviposition cages. See Table 4 for complementary data.

The BSF+AD treatment had significant effects on *Cmac* growth and development (Table 3). *Cmac* larvae fed BSF+AD required more time to metamorphose into pupae than those fed BSE+CM. Larvae fed BSF+AD had lower rates of survival to pupal and adult stages than those fed BSE+CM. Similarly, teneral adults had less body mass when fed BSF+AD than BSE+CM (Table 3). Irrespective of diet, the days required for *Cmac* larvae to metamorphose into pupae was negatively correlated with the body mass of emerged adults (Figure 2a; *r* = −0.820, *p* = 0.00002, *N* = 19); a longer development time correlated with the emergence of smaller-sized adults.

The time necessary for newly emerged adults to commence mating (i.e., *in-copula* pairing of males and females) was significantly longer for those fed BSF+AD than BSE+CM (Table 4). The time (days) between emergence and mating was not correlated with the number of egg clutches eventually produced by mated females (Figure 2b; *r* = 0.328, *p* = 0.147, *N* = 21). The time between the onset of mating to the production of the first egg clutch was not affected significantly by treatments (Table 4). Regardless of diet, the onset of mating to the first egg clutch was negatively correlated with egg clutch production per female within the 30-day evaluation period (Figure 2c; *r* = −0.490, *p* = 0.024, *N* = 21). Finally, the number of clutches produced by females did not differ between treatments. Similarly, the number of eggs within a clutch did not differ between treatments (Table 4).

## 4. Discussion

The observation that a diet composed of BSF, BSF+CM, or BSF+AD was less effective than BSE+CM, a standard in-house diet, for *Cmac* growth and development but was generally suitable for *Cmac* oviposition could suggest that developing larvae had difficulty ingesting and processing BSF. Although all diets were pulverized into a powder formulation, the particle size of BSF larval powder was slightly larger than the BSE powder in this study. Diet particle size was not measured. However, in recent work, wheat bran, chicken feed pellet, or ground corn kernel diets of a particle size of less than 2.0 mm enhanced the growth of yellow mealworm (*T*. *molitor*) larvae [41]. Due to their small size, i.e., small mouthparts, it is conceivable that *Cmac* first instars were incapable of obtaining sufficient food, thus limiting their growth and development. Conceivably, due to their larger size, *Cmac* adults would not have difficulty consuming BSF larval powder and obtaining the sufficient nutrients necessary for reproduction. Regrettably, data to support this assertion was not collected in this study. Nevertheless, research has demonstrated that *Cmac* larval and adult stages can have slightly different nutritional requirements [42].

We also note that treatment formulations of BSF used in these experiments could have been a factor that affected *Cmac* development. A diet composed of 100% BSF or 90% BSF (combined with 5% green algae and 5% myristic acid) in this study could have contained too much saturated fat. BSF larvae contain more saturated fats (including 21–37% lauric acid, of the total fatty acids) than other insects, e.g., yellow mealworms (0.2–1.3% lauric acid), used in the feed industry [43]. Moreover, the BSE used in this study contained only 7.3% fatty acid content, according to the product label. *Cmac* larvae could have had difficulty digesting or assimilating lauric acid or other fatty acids in the BSF diets. Alternatively, the high saturated fat content could have interfered with the digestion of protein (amino acids). As a possible remedy, a lower proportion of BSF powder could be used in diet mixtures for *Cmac* larvae. BSF quantities, such as 5, 10, 20, and 40%, in diet mixtures could be tested in a future study. No prior studies have tested any formulations of BSF in diets for predatory insects, to our knowledge. However, one study tested several formulations of BSF hemolymph in yeast extract and egg yolk-based diets for the predatory mite *A*. *swirskii* [22]. The authors discovered that a formulation containing no more than 20% BSF larval hemolymph in the diets was most suitable for *A*. *swirskii* growth, development, and reproduction.

Research on supplementing diets for farm animals has revealed that inclusion rates of no more than 25% BSF larval powder were the most effective. In a study involving rainbow trout, diets containing up to 25% protein from BSF mature larvae (as a replacement for fishmeal) did not negatively affect growth performance and quality of rainbow trout *Oncorhynchus mykiss* (Walbaum) (Salmoniformes: Salmonidae) [44]. Moreover, adding 10.5% dried BSF larvae to a fishmeal-based diet improved the growth performance of juvenile Pacific white shrimp *Litopenaeus vannamei* (Boone) (Decapoda: Penaeidae) [45]. 

The time (days) for *Cmac* to metamorphose into pupae was correlated with the body mass of adults, in both experiments, suggesting that there was a carryover effect of diet from immature to adult stages. Diets containing BSF resulted in smaller adults as clearly illustrated in Figure 1a and Figure 2a. Production of smaller-sized adults in coccinellid species, and other coleopteran predators, is often a direct consequence of a decrease in food (diet) quantity or quality available during pre-imaginal development [46,47,48]. Also, adults reared on diets containing BSF were somewhat reluctant to commence mating (see Table 2 and Table 4) in both experiments, further suggesting a carryover effect of the diet.

The onset of mating to laying the first egg clutch by *Cmac* females was significantly correlated with the total production of egg clutches within the 30-day observation period, regardless of diet treatment (see Figure 1c and Figure 2c), which suggests that females that delayed ovipositing were less capable of producing their expected number of clutches. The average pre-oviposition period of mated *Cmac* females was 12 days when reared on a BSE powder plus 5% palmitic acid diet in a previous study [33]. This diet significantly increased the number of egg clutches, but not eggs per clutch, laid by *Cmac* females [33]. Finally, the total production of egg clutches did not differ significantly between treatments in both experiments in this study, suggesting that the nutritional composition in the BSF diets did not affect *Cmac* oviposition responses, even though adults were smaller in body size. Therefore, a mixed diet containing high proportions of BSF does not hamper egg clutch production in *Cmac* females.

## 5. Conclusions

In conclusion, BSF larval powder has the potential to be food that supports the oviposition behavior of *Cmac* adults. Using the in-house diet (BSE+CM) for *Cmac* larvae but BSF alone or BSF+AD for adults could be a cost-effective option. BSF dried larvae have been sold for approximately 24.00 USD per pound, i.e., 53.33 USD per kilogram (Symton^®^ Black Soldier Fly; https://symtonbsf.com, assessed on 19 October 2023). In contrast, freeze-dried, decapsulated BSE have been sold for approximately 49.90 USD per pound, i.e., 110.01 USD per kilogram (Brine Shrimp Direct Inc., Ogden, UT, USA, https://www.brineshrimpdirect.com; assessed on 18 October 2023). Since BSF is currently two-fold less expensive than BSE, it could be a good financial investment to incorporate BSF into a *Cmac* rearing system. Further research is needed to evaluate various mixtures of BSF, BSE, and AD, with or without CM, to develop an ideal formulation that supports *Cmac* immature growth, development, and reproduction over multiple generations.

## Figures and Tables

**Table 1:** Mean ± SE number of *Cmac* first instars surviving to pupal and adult stages, days to metamorphose into pupae, and adult body mass estimates in replicate communal arenas. Diet treatments included brine shrimp egg powder plus *Chlorella vulgaris* algae and myristic acid (BSE+CM), black soldier fly larval powder (BSF), or a novel mixture (BSF+CM).

^1^ Diet Treatments	First Instars per Arena	Days to Pupal Stage	Pupae	Adults	Adult Body Mass (mg)
BSE+CM	10	13.08 ± 0.14 b	8.80 ± 0.39 a	8.70 ± 0.37 a	15.29 ± 0.37 a
BSF	10	17.48 ± 0.17 a	5.50 ± 0.43 b	5.20 ± 0.47 b	9.24 ± 0.23 c
BSF+CM	10	17.87 ± 0.18 a	6.80 ± 0.55 b	6.30 ± 0.58 b	10.16 ± 0.20 b
*F*	--	267.63	12.93	13.99	139.78
*df*	--	2, 27	2, 27	2, 27	2, 27
*p*	--	<0.001	<0.001	<0.001	<0.001

^1^ Diet treatments are described in the Materials and Methods. Sample size: 10 *Cmac* first instar larvae in 10 replicate Petri dish arenas per diet treatment at the onset of the experiment. The Petri dish arena was the sampling unit for statistical analyses. Mean ± SE values followed by a different letter in a column are significantly different (*p* < 0.05; Holm–Sidak test).

**Table 2:** Mean ± SE number of days from *Cmac* adult emergence to mating, mating to laying first egg clutch, and mean ± SE number of total clutches and eggs per clutch oviposited within 30 days. Diet treatments included brine shrimp egg powder plus *Chlorella vulgaris* algae and myristic acid (BSE+CM), black soldier fly larval powder (BSF), or a novel mixture (BSF+CM).

^1^ Diet Treatments	*N*, Females	Emergence to Mating (Days)	Mating to First Egg Clutch (Days)	Clutches per Female	Eggs per Clutch
BSE+CM	13	8.38 ± 0.42 b	8.93 ± 1.71 a	3.69 ± 0.79 a	14.80 ± 2.22 a
BSF	7	14.0 ± 1.46 a	13.43 ± 3.12 a	4.00 ± 1.33 a	6.35 ± 0.95 b
BSF+CM	8	11.0 ± 0.96 ab	12.50 ± 3.66 a	4.37 ± 1.05 a	9.62 ± 1.05 ab
*F*	--	10.79	0.89	0.12	5.09
*df*	--	2, 25	2, 25	2, 25	2, 25
*p*	--	<0.001	0.42	0.88	0.014

^1^ Diet treatments are described in the Materials and Methods. The sample sizes (*N*) were 13, 7, and 8 *Cmac* newly emerged females, with mates, per respective treatment (as given above) in oviposition cages. The oviposition cage was the sampling unit for statistical analyses. The oviposition response period was restricted to 30 days, commencing after the first mating observation. Mean ± SE values followed by a different letter in a column are significantly different (*p* < 0.05; Holm–Sidak test).

**Table 3:** Mean ± SE number of *Cmac* first instars surviving to pupal and adult stages, days to metamorphose into pupae, and adult body mass estimates in replicate communal arenas. Diet treatments included BSE+CM and a novel mixture of black soldier fly plus an artificial diet (BSF+AD).

^1^ Diet Treatments	First Instars per Arena	Days to Pupal Stage	Pupae	Adults	Adult Body Mass (mg)
BSE+CM	10	14.01 ± 0.27 b	9.00 ± 0.33 a	8.60 ± 0.40 a	14.59 ± 0.32 a
BSF+AD	10	17.23 ± 0.23 a	6.56 ± 0.44 b	6.22 ± 0.55 b	11.17 ± 0.40 b
*t*	--	8.91	4.46	3.56	6.71
*df*	--	17	17	17	17
*p*	--	<0.001	<0.001	0.002	<0.001

^1^ Diet treatments are described in the Materials and Methods. Sample size: 10 *Cmac* first instar larvae in 10 replicate Petri dish arenas per BSE+CM treatment and in 9 replicate Petri dish arenas per BSF+AD treatment at the onset of the experiment. The Petri dish arena was the sampling unit for statistical analyses. Mean ± SE values followed by a different letter in a column are significantly different (*p* < 0.05; Holm–Sidak test).

**Table 4:** Mean ± SE number of days from *Cmac* adult emergence to mating, mating to laying first egg clutch, and mean ± SE number of total clutches and eggs per clutch oviposited within 30 days. Diet treatments included BSE+CM and a novel mixture of black soldier fly plus an artificial diet (BSF+AD).

^1^ Diet Treatments	*N*, Females	Emergence to Mating (Days)	Mating to First Egg Clutch (Days)	Clutches per Female	Eggs per Clutch
BSE+CM	12	6.33 ± 0.61 b	8.58 ± 2.12 a	4.50 ± 0.73 a	11.05 ± 1.73 a
BSF+AD	9	11.33 ± 1.44 a	13.00 ± 2.07 a	6.22 ± 1.28 a	11.23 ± 1.40 a
*t*	--	3.51	1.45	1.24	0.08
*df*	--	19	19	19	19
*p*	--	0.002	0.16	0.23	0.94

^1^ Diet treatments are described in the Materials and Methods. The sample sizes (*N*) were 12 and 9 *Cmac* newly emerged adult females, with mates, per respective treatment (as given above) in oviposition cages. The oviposition cage was the sampling unit for statistical analyses. The oviposition response period was restricted to 30 days, commencing after the first mating observation. Mean ± SE values followed by a different letter in a column are significantly different (*p* < 0.05; Holm–Sidak test).

## Data Availability

Datasets representing the results presented in this study can be made available on ResearchGate by the senior author.
